# From Wheelchair Bound to Working: A Case Study of Intravenous Ketamine Infusions in Treating Stiff Person Syndrome

**DOI:** 10.7759/cureus.59397

**Published:** 2024-04-30

**Authors:** Ashraf F Hanna, Danielle Bolling, Mariam Tadros

**Affiliations:** 1 Pain Management/Anesthesiology, Florida Spine Institute, Clearwater, USA; 2 Pain Management, University of South Florida (USF) Health Morsani College of Medicine, Tampa, USA; 3 Pain Management, Lake Erie College of Osteopathic Medicine, Bradenton, USA

**Keywords:** intravenous ketamine, treatment-resistant, pain management, chronic pain, ketamine hydrochloride, stiff person spectrum disorder, stiff person syndrome

## Abstract

Stiff Person Syndrome (SPS) is a rare autoimmune condition marked by extremely painful muscle spasms, stiffness, and rigidity throughout the body. Its rarity often translates to limited treatment options for patients and, occasionally, challenges in obtaining a definitive diagnosis. SPS also impacts patients' mental health, social and economic involvement, and overall quality of life.

A 43-year-old man was initially being seen for lumbar radicular pain. A clinical diagnosis of SPS was made by a neurologist and confirmed by in-clinic follow-ups and anti-glutamic acid decarboxylase (anti-GAD) antibody testing. The Pain Management doctor agreed with this diagnosis and offered intravenous (IV) ketamine treatment, which he has found to positively impact the treatment of similar disorders. After an initial 10-day infusion, the patient reported improvement in pain and function. For almost two years, the patient received intravenous immunoglobulin (IVIg) and IV ketamine treatments to manage their condition and maintain pain control as well as quality of life. When the patient's symptoms began worsening after IVIg infusions, the decision to withdraw IVIg infusions and continue ketamine infusions was made. After discontinuing IVIg infusions, the patient reported improvement in function and pain level and continues to receive monthly two-day ketamine boosters. Outside of the infusions, the patient was able to discontinue the use of fentanyl patches and continued taking ketamine lozenges, oxycodone-acetaminophen, and dextromethorphan for at-home pain management. The patient's symptoms continue to be managed effectively with their current regimen, enabling their return to work and experiencing an enhanced quality of life.

This case illustrates the potential benefits of IV ketamine treatment for patients with treatment-resistant SPS and similar neurologic and autoimmune disorders. Understanding and examining treatment alternatives for rare syndromes is crucial for achieving optimal patient outcomes. Additionally, documenting such cases offers valuable insights into the mechanism of ketamine, extending beyond these syndromes.

## Introduction

Stiff Person Spectrum Disorder (SPSD) is a rare, progressive neurological and autoimmune disorder. The patient studied in this report has been diagnosed with Stiff Person Syndrome (SPS), which belongs to the group of SPSDs. SPS, also known as Moersch-Woltman syndrome or formerly "Stiff Man Syndrome," has an estimated prevalence of approximately one in every million individuals [1]. Despite being extremely rare, patients who suffer from Stiff Person Syndrome must not be overlooked. Primary symptoms of SPS are associated with the dysfunction of inhibitory mechanisms within the central nervous system (CNS) [2]. The brain and spinal cord comprise the CNS, which is responsible for the intake and processing of sensory information and the output of motor signals.

The cause of SPSD is thought to be idiopathic, but current studies suggest it to be an autoimmune response against the inhibitory pathways controlled by the neurotransmitter called gamma-aminobutyric acid (GABA). In addition to physically presenting symptoms, an association between SPS and anti-glutamic acid decarboxylase (anti-GAD) antibodies is present in up to 80% of classic SPS cases [3]. Glutamic acid decarboxylase (GAD) is an enzyme involved in the production of GABA, which controls muscle activity [1,2]. GABA is an inhibitory neurotransmitter in the brain and slows the brain by blocking action potentials in the CNS, thus decreasing the action of neurons in the brain [4]. Anti-GAD antibodies have an inhibitory effect on GABA synthesis. For patients with SPS, the presence of high anti-GAD antibodies causes the overstimulation of neurons, which leads to intense and painful muscle spasms.

Primary symptoms of SPS include fluctuating muscle rigidity, painful muscle spasms, abnormal posture and gait, and hypersensitivity to stimuli such as noises, physical contact, and stressful situations [5,6]. Diagnosis involves a comprehensive assessment, including a physical examination, a review of medical history, and specific laboratory tests such as anti-GAD antibodies and electromyography (EMG). As a progressive condition with limited treatment options, untreated SPS can culminate in full-body rigidity and respiratory compromise, possibly leading to death by respiratory arrest [7,8]. In a longitudinal study involving 57 patients diagnosed with SPS, 46 out of 57 (approximately 80%) lost the ability to walk unassisted as their condition advanced. These patients then require a wheelchair for ambulation or must remain bed-bound due to disability. Despite the utilization of symptomatic treatments, the study concluded that SPS displays a progressive nature, resulting in severe disability over time [9]. The prognosis for patients with SPSDs remains uncertain due to limited data, making it challenging to predict the likelihood of specific groups developing SPSDs or the stability of symptoms.

Ketamine is a drug that has been used as a human anesthetic since the 1960s. It gained popularity due to its unusual effect on the body, allowing cardiorespiratory stability while at the same time causing sedation and analgesia. Ketamine is a relatively safe anesthetic and works very well as an analgesic (medication to relieve pain) [10]. Ketamine is water and lipid-soluble, which allows for the administration by various routes, including intravenous (IV), intramuscular (IM), oral, nasal, rectal, subcutaneous, and epidural [10]. In recent years, the potential role of ketamine in the treatment of psychological and neurological disorders has emerged. Despite being indicated as a dissociative anesthetic, ketamine has proven to be an effective off-label treatment for various complex conditions found to be resistant to conventional therapies [11].

The most common ketamine-related side effects include hypersalivation, hyperreflexia, dissociation, nausea, visual distortions, hallucinations, difficulty speaking, and numbness [10-12]. None of these side effects observed by the National Institute of Health (NIH) Clinical Center lasted longer than four hours and none had long-lasting effects. The side effects observed coincided with the use of an intravenous subanesthetic dose of ketamine [12].

The addition of dextromethorphan, which acts as a N-methyl-D-aspartate receptor (NMDAR) antagonist, has been shown to alleviate pain and provide further relief when given in combination with ketamine [13,14]. NDMARs are located in the central and peripheral nervous system, and evidence suggests that NMDARs in both areas play a role in pain perception. The use of an NMDAR antagonist is limited by its side effects. Side effects related to the central nervous system include memory impairment, hallucinations, nightmares, delirium, ataxia (poor muscle control), and motor incoordination [15]. When combining dextromethorphan with ketamine, it's crucial to closely monitor for any adverse effects on the patient. Particularly in individuals with muscular and nervous system disorders, dextromethorphan may not be appropriate.

## Case presentation

Chronic illness affects various aspects of patients' lives. The patient profiled in this case experienced physical, social, and economic challenges as his condition advanced. He lost the ability to work, and before intravenous ketamine treatments, he was wheelchair-bound. The emotional strain endured by patients with Stiff Person Syndrome can precipitate severe depression and anxiety, profoundly impacting their relationships and overall quality of life. A board-certified anesthesiologist and pain management doctor from the Florida Spine Institute found that IV ketamine infusions have helped others with similar symptoms and disorders and decided to initiate treatment on this patient. Prior to treatment initiation, he described his condition as "debilitating" and articulated a sense of losing the ability to lead a fulfilling life. However, with the initiation and progression of treatment, both his attitude and symptoms improved significantly.

In March 2020, a 43-year-old male, initially evaluated in the clinic for lumbar pain in 2017, presented to a pain management clinic following a recent diagnosis of Stiff Person Syndrome. The patient complained of having no relief of pain or related symptoms while taking his current medication regimen. All pain scores were captured by administering a standard unipolar visual analog scale (VAS) for pain, and he reported that without any pain medication, his pain level ranged from 8/10 to 10/10. His past surgical history consisted of procedures such as L5-S1 fusion, hernia repair, right knee arthroscopy for a meniscal tear, right labrum repair, and cholecystectomy. Additionally, his previous diagnoses included chronic pain syndrome, cystic fibrosis, cervical dystonia, and chronic headaches. At that time of presentation, he required a wheelchair for ambulation. Given the patient's symptom progression and bloodwork, the diagnosis of Stiff Person Syndrome was supported by pain management specialists at the Florida Spine Institute. Intravenous ketamine infusions were offered to this patient to alleviate some of his pain and calm the muscle spasms and neuropathy that he was experiencing. While awaiting IV ketamine treatment, the patient was started on ketamine lozenges, in which he then reported 40% pain relief. Prior to commencing the infusions, he described his pain level as 8/10 and characterized the muscle spasms and stiffness as "debilitating," significantly affecting his daily activities and quality of life.

The patient began a 10-day IV ketamine infusion according to protocol on July 27, 2020, at The Florida Spine Institute. The starting dose was 200 mg of ketamine hydrochloride, 410 mg of lidocaine, 4000 mg of magnesium sulfate, 4 mg of midazolam, and 8 mg of ondansetron in a 100 mL bag of saline infused over four hours. The ketamine dosage was increased from 200 mg to 400 mg on day two and remained at 400 mg through day 10. All doses were well tolerated, and no unexpected adverse effects were observed. VAS pain scores were documented at each clinic visit, with the exception of two interim infusion days. Throughout the initial 10-day infusion period, the patient reported fluctuations in his pain level, varying between scores of 3 to 8.

Upon returning for the second day of infusions, he reported his pain level as unchanged (score 8/10) but stated he had decreased spasms, which became more "manageable." On the morning of his third infusion, he reported his pain to be "tolerable," rating it as 7/10, and noted improved sleep quality. Following the infusion on the third day, he experienced his lowest pain level (score 3/10), which gradually increased afterward during the night while lying in bed.

About seven weeks later, at an office visit on September 17, 2020, the patient demonstrated the ability to ambulate with a cane instead of relying on a wheelchair. Initially, his pain was reported to be between 8/10 to 10/10 prior to the ketamine infusions. However, during this visit, he indicated experiencing 50-70% pain relief as a result of the ketamine treatments, stating his pain level to be on average, around 4/10. During an office visit to Florida Spine Institute on December 16, 2020, the patient excitedly expressed his intention to resume work as a general contractor. Remarkably, treatment with IV ketamine over the past five months has significantly ameliorated his symptoms. He also reported that since initiating the treatments, his mental clarity had improved nearly 100%.

On March 16, 2021, the decision was made to discontinue the fentanyl 25 mcg/hr patch and increase ketamine lozenges to 50 mg every six hours. The patient has not required restarting the fentanyl patches since this modification. In addition, he was previously consuming oxycodone-acetaminophen 10-325 mg four times daily before treatment initiation, and there has been no necessity for a dosage escalation during the course of treatment. Over the next months, the patient’s pain was reported to be well controlled with monthly IV ketamine boosters. The most severe instances of pain and muscle spasms coincided with reports of increased stress.

On December 6th, 2021, he noted experiencing 90% relief from his prior infusion, which lasted for one week until he underwent his intravenous immunoglobulin (IVIg) treatment, resulting in a flare-up of pain. Shortly after, on a visit to the University of South Florida (USF) Neurology on December 16, 2021, anti-GAD antibody levels (sample taken 09/13/2021) were elevated at 489 units/ml (reference range 0-5). According to USF Neurology, the patient underwent IVIg treatment every three months, which he had previously credited with significantly reducing spasm episodes prior to this visit. The patient conveyed to USF Neurology that his recent IVIg treatment resulted in a setback of about 30-35%. Subsequently, he experienced difficulties with walking once again. It was only after receiving IV ketamine infusions that he reported a restoration of function and pain relief.

On April 21, 2022, USF Neurology reported the patient discontinued IVIg treatment due to worsening symptoms and an associated increase in anti-GAD antibody levels. The decision to continue IV ketamine treatment with Florida Spine Institute was made at this time. After discontinuing IVIg treatment, the patient’s self-reported quality of life scores became more stable.

**Figure 1:**
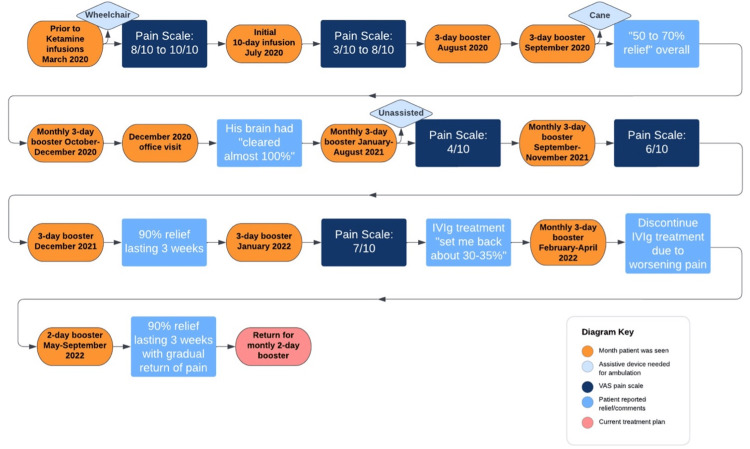
Pain scale and reported relief throughout treatment

**Figure 2:**
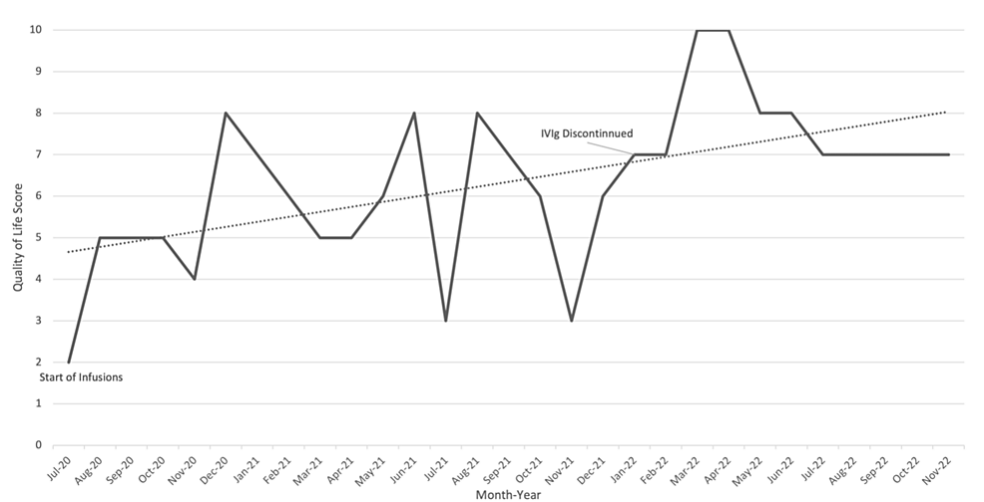
Quality of life scores reported by the patient Quality of life scores were reported using the American Chronic Pain Association’s Quality of Life Scale: A Measure Of Function For People With Pain. A score of 0 indicates the patient is “Non-functioning” and is described as “Stay in bed all day. Feel hopeless and helpless about life.” When the patient began IV ketamine treatments, he reported a quality of life score of 2, described as “Get out of bed but don’t get dressed. Stay at home all day.” At a 6, the patient reports the ability to “Work/volunteer limited hours. Take part in limited social activities on weekends.” At a score of 10, the patient has a “Normal Quality of Life” and reports the ability to “Go to work/volunteer each day. Normal daily activities each day. Have a social life outside of work. Take an active part in family life.”

The patient continues to return to the clinic for three-day boosters monthly. Figure 1 highlights the pain scale as well as a timeline of interventions and improvements that the patient experienced throughout his treatment. Liver function is evaluated twice a year to monitor both liver function and drug metabolism. During his latest appointment, he still demonstrated the ability to ambulate without the aid of assistive devices and reported that his quality of life had stabilized (Figure 2). No ketamine-related side effects or physical dependence have been observed during treatment.

## Discussion

The patient evaluated in this case report initially received ketamine and anti-GAD antibody infusions (IVIg). Although anti-GAD antibodies are commonly administered to patients with SPS exhibiting elevated GAD antibodies, there is no established protocol for treatment if a patient ceases to respond to these infusions, as observed in this case report. Fortunately, prior to this unanticipated reaction, the patient had been undergoing ketamine infusions. Consequently, when the anti-GAD antibody infusions were withdrawn, he was able to continue with the ketamine infusions. Ketamine is gaining traction as an effective treatment for alleviating symptoms associated with neurological and autoimmune disorders, demonstrating notable efficacy. The patient under examination in this report experienced substantial symptom relief in all aspects of his disease, including pain, muscle rigidity and spasms, mental clarity, and depression with ongoing ketamine therapy. He can now walk unassisted and has successfully returned to work.

Throughout this patient’s treatment journey, he transitioned from relying on wheelchair assistance to walking independently without the need for any assistive device. Remarkably, no ketamine-related side effects or signs of physical dependence have been noted during the course of treatment, underscoring the safety and effectiveness of ketamine as both an analgesic and a therapeutic intervention for symptoms such as muscle spasms and cramping associated with muscular and autoimmune disorders. The patient's quality of life and ambulatory function have seen remarkable enhancements with the integration of IV ketamine into his treatment regimen.

Returning for monthly boosters requires a significant amount of commitment from patients and their caregivers. Ketamine infusions alone could not provide the desired relief for this patient, and the addition of at-home ketamine lozenges 60 mg every six hours as needed, dextromethorphan 50 mg three times daily as needed, ondansetron 4 mg every six hours as needed has shown to provide the greatest amount of relief. Dextromethorphan is used to supplement pain relief and duration of relief. Because dextromethorphan is important for normal central nervous system (CNS) function, further investigation into the effects of dextromethorphan on patients with SPS could provide insight into the pathophysiology of the disorder. It is also important to note that the use of dextromethorphan has not presented any related side effects.

The addition of other medications during the infusion process serves various purposes. Midazolam is used to reduce or prevent psychotomimetic side effects that ketamine can cause in some patients. These side effects include both auditory and visual hallucinations [16]. Zofran (ondansetron) is used to treat nausea that may be associated with the patient’s condition or as a side effect of the medications being used. Lidocaine infusion has been found to be effective in the short-term treatment of various neurologic and manual disorders [17]. The addition of magnesium sulfate to intravenous ketamine infusion is used to decrease and prevent neuropathic pain [13]. It is believed that the efficacy of this addition is reliant on the effects of magnesium sulfate as an N-methyl-d-aspartate receptor (NMDAR) modulator. NMDARs are involved in the development of neuropathic pain [15].

When examining a patient’s case through recorded pain scales, medication usage, and laboratory findings, it is not always easy to gain a comprehensive understanding of the patient's quality of life. In the case of this patient, it's crucial to not only evaluate their clinical progress but also to assess how their treatment has influenced their daily life. Chronic pain patients are at a substantially higher risk for social isolation due to limited mobility, poor mental health, and physical limitations. These associations have been found to be bidirectional, indicating that social isolation can predict how pain impacts carrying out daily activities, pain perception, physical health, and mental health [18,19]. Notably, this patient has been able to actively participate in society as a contractor and reintegrate himself into the workforce. Furthermore, he has managed to sustain meaningful human connections and uphold his social and mental well-being. 

Conditions such as SPS present challenges in data collection due to their low prevalence. Reporting treatments and outcomes becomes increasingly vital to construct a more comprehensive understanding of the disorder. Moreover, the uncertainties surrounding the pathogenesis of SPS contribute to the complexity of diagnosis and treatment, further underscoring the need for continued research and reporting in this field. Supplementary case reports must be published on patients with Stiff Person Spectrum Disorders to refine disease characteristics and share potential treatments. As a rare disease, patients with SPS encounter limited treatment options and insufficient data on treatment effectiveness. Therefore, the publication of cases like this one holds increasing significance in shedding light on the impact of treatments on patients' quality of life with SPS.

When addressing treatment-resistant conditions, it is crucial to consider the multitude of expenses incurred from pursuing various treatments and managing daily life with these conditions. For many individuals, coping with these conditions has resulted in job loss and the loss of social support networks, leading to a substantial decrease in quality of life [20]. The lack of structural support leads to a vicious cycle that many chronic pain patients and patients with treatment-resistant conditions fall into. It begins with the onset of their condition, many struggle to find a diagnosis or face misdiagnosis. For certain patients, like the one discussed in this report, their conditions may initially respond well to treatment, allowing them to sustain a semblance of normalcy. However, as their condition advances, they may experience job loss, strain on relationships, financial difficulties, and social isolation, all of which profoundly impact their overall well-being [20]. This is where Florida Spine Institute meets many of its patients, and unfortunately, providing care during this period is challenging. As previously outlined, patients encounter a multitude of obstacles to accessing care by the time they need these treatments. It's evident that without emotional, financial, physical, and structural support, this vicious cycle can ensue. The patient discussed in this report experienced success primarily due to his robust social support network and the established rapport with his treating physician. Although his condition led to a hiatus from work for approximately 9 months, his support system and treatment regimen enabled him to receive consistent care, facilitating a prompt return to work when feasible, a prospect that brought him satisfaction and immense joy.

Addressing barriers to care, such as transportation limitations, caregiver shortages, social isolation, economic hardships, and other related challenges faced by patients with disabilities, necessitates a collaborative effort involving policymakers, public health experts, and insight from patient experiences.

Far too frequently, academic research overlooks the perspective and voice of the patient. With the aim of safeguarding privacy, we have inadvertently silenced individuals whose narratives extend beyond mere clinical outcomes, narratives that possess the power to enlighten and motivate others. The case report necessitates a patient who consents to share their personal journey, a decision that entails diminishing a degree of privacy. For certain individuals whose illnesses dominate their lives, the desire to positively influence others in similar circumstances drives them to share their stories. Through this patient's narrative, he can redefine his identity beyond being merely a patient with a medical condition [21].

The writing and publication of case reports are growing in significance, particularly in the exploration of new innovative treatments and off-label utilization of medications such as IV ketamine. Case reports offer several implications, including advancements in personalized medicine, the introduction of fresh and innovative concepts, the rapid dissemination of knowledge to a broad audience, and the empowerment of patients to contribute their perspectives to the medical community [21]. Case reports such as this facilitate the integration of public health principles, artistic elements, personal narratives, and opinions with the scientific foundations that make up high-quality research.

## Conclusions

SPS is a rare disease that presents with several different manifestations and may be overlooked by healthcare providers. In this case, we described a 43-year-old male who was diagnosed with Stiff Person Syndrome and, at that time, had no relief in symptoms with his medication regimen. Initiating treatment with IV ketamine infusions alleviated his pain, increased his mobility, and improved his mental health and overall quality of life. In general, treatment options for SPS are limited, but IV ketamine infusion proves to be a hopeful option for patients living with this disease. Hope remains that future clinical trials will substantiate ketamine's efficacy in treating neurological and autoimmune disorders like Stiff Person Syndrome.
